# Factors associated with performing tuberculosis screening of HIV-positive patients in Ghana: LASSO-based predictor selection in a large public health data set

**DOI:** 10.1186/s12889-016-3239-y

**Published:** 2016-07-13

**Authors:** Susanne Mueller-Using, Torsten Feldt, Fred Stephen Sarfo, Kirsten Alexandra Eberhardt

**Affiliations:** Bernhard Nocht Institute for Tropical Medicine, Bernhard Nocht Str. 74, 20359 Hamburg, Germany; Hamburg Institute of International Economics, Heimhuder Str. 71, 20148 Hamburg, Germany; Clinic of Gastroenterology, Hepatology and Infectious Diseases, University Hospital of the Heinrich Heine University Duesseldorf, Moorenstr. 5, 40225 Duesseldorf, Germany; Kwame Nkrumah University of Science and Technology, University Post Office - KNUST, Kumasi, Ghana; Komfo Anokye Teaching Hospital, P.O. Box 1934, Kumasi, Ghana

**Keywords:** LASSO, Variable selection, HIV/AIDS, Tuberculosis screening, Sub-Saharan Africa

## Abstract

**Background:**

The purpose of this study is to propose the Least Absolute Shrinkage and Selection Operators procedure (LASSO) as an alternative to conventional variable selection models, as it allows for easy interpretation and handles multicollinearities. We developed a model on the basis of LASSO-selected parameters in order to link associated demographical, socio-economical, clinical and immunological factors to performing tuberculosis screening in HIV-positive patients in Ghana.

**Methods:**

Applying the LASSO method and multivariate logistic regression analysis on a large public health data set, we selected relevant predictors related to tuberculosis screening.

**Results:**

One Thousand Ninety Five patients infected with HIV were enrolled into this study with 691 (63.2 %) of them having tuberculosis screening documented in their patient folders. Predictors found to be significantly associated with performance of tuberculosis screening can be classified into factors related to the clinician’s perception of the clinical state, as well as those related to PLHIV’s awareness. These factors include newly diagnosed HIV infections (*n* = 354 (32.42 %), aOR 1.84), current CD4+ T cell count (aOR 0.92), non-availability of HIV type (*n* = 787 (72.07 %), aOR 0.56), chronic cough (*n* = 32 (2.93 %), aOR 5.07), intake of co-trimoxazole (*n* = 271 (24.82 %), aOR 2.31), vitamin supplementation (*n* = 220 (20.15 %), aOR 2.64) as well as the use of mosquito bed nets (*n* = 613 (56.14 %), aOR 1.53).

**Conclusions:**

Accelerated TB screening among newly diagnosed HIV-patients indicates that application of the WHO screening form for intensifying tuberculosis case finding among HIV-positive individuals in resource-limited settings is increasingly adopted. However, screening for TB in PLHIV is still impacted by clinician’s perception of patient’s health state and PLHIV’s health awareness. Education of staff, counselling of PLHIV and sufficient financing are needed for further improvement in implementation of TB screening for all PLHIV. The LASSO approach proved a convenient method for automatic variable selection in a large public health data set that requires efficient and fast algorithms.

**Trials registration:**

ClinicalTrials.gov NCT01897909 (July 5, 2013).

## Background

Recent advances in data collection and storage techniques in public health research often result in large data sets with sometimes even more variables than observations, a phenomenon that in statistics is often termed as the “large p and small n problem” [1]. Finding relevant associations between variables often appears as searching for a needle in the haystack. Usually these data do not only include various clinical information, but also demographical and socio-economical details as well as behavioural, nutritional or geographical data with many of these variables being correlated.

Detecting relevant predictors for an outcome using conventional explorative data analysis frameworks, such as running univariate regression tests for each potential preventive or risk factor and thereafter including those parameters with *p* < 0.05 into a multivariate regression model using forward or backward variable selection is time-consuming for large data sets. In addition, it often results in high numbers of determined predictors if collinearities have not been reasonably addressed [2]. Many risk or preventive factors are also not convenient for translating evidence into public health practice because clinicians are usually short of time and unable to consider more than a handful parameters for decision-making during routine work [3]. They rather rely on their individual clinical experience than on evidence-based interrelationships if these are too complex [4].

The Least Absolute Shrinkage and Selection Operators procedure (LASSO) offers a possible solution to these problems. Developed in 1996 by Tibshirani, and initially mainly used in data mining, the LASSO’s objective function penalizes the absolute size of the regression coefficients and drives the coefficients of irrelevant variables towards zero, which results in automatic variable selection [5]. By using the advantages of subset selection and ridge regression, the LASSO approach enables for statistical modelling that is robust and allows for easy interpretation.

We are interested in developing a predictive modelling framework that links (at least partly correlated) demographical, socio-economical, clinical and immunological variables to a public health outcome. Specifically, we focus on developing a model that relates the performance of screening for co-infection with *Mycobacterium tuberculosis* in patients with Human Immunodeficiency Virus (HIV) infection to associated factors in Ghana, West Africa. Exploring which factors are related to conduction and non-performance of tuberculosis (TB) screening by health care providers is of high importance for national and international health services, because TB remains the most important opportunistic infection in people living with HIV (PLHIV) and is the leading cause of mortality and morbidity among them across sub-Saharan Africa, including Ghana [6]. If detected, TB is normally curable by a specific combination of antibiotics and by treating HIV with combination antiretroviral therapy (HAART) [7, 8]. In Ghana, an estimated 20,000 people developed an active TB only in 2011, of which 22 % where not detected and the mortality rate remains high at about 7.5 per 100,000. To address the low case detection rate, Ghana developed a National Tuberculosis Health Sector Strategic Plan, which includes an intensified case finding program among PLHIV [9]. A higher detection rate of TB among PLHIV should not only decrease the burden of co-infected HIV-positive individuals, but also lower the rate of new TB infections among PLHIV and protect hospital staff working at HIV outpatient departments. In order to simplify TB screening, the World Health Organization (WHO) has introduced a standardised screening form that consists of symptom-oriented questions for resource-limited settings. If one question is answered affirmatively, subjects are considered to be “TB suspects” and further diagnostics (sputum testing, chest X-ray) are initiated [9]. This screening tool should help implementing TB screening of HIV-positive patients in treatment facilities as a standard procedure and increase the detection rate of TB cases [10–12]. However, the rate of non-detected cases of co-infections with TB among PLHIV in Ghana remains high and the underlying causes are not clear to date [9, 13]: Are PLHIV who have been on treatment for a longer period less likely to be screened for TB because the WHO screening form has been introduced only a few years ago? Or do these patients have a higher chance than more recently diagnosed patients that screening has taken place at any of their multiple clinical visits? Do factors, such as age, gender or religion have an impact on performance of screening for TB? Are patients with a higher education and higher income more likely to be screened for TB than less educated patients? Is there evidence that procedures in HIV outpatient departments are still not throughout standardised and therefore, TB screening depends on individual staff?

This paper has both, a methodological and an empirical objective. The methodological aim is to propose the LASSO method as a convenient tool for explorative data analysis, variable selection and model interpretation in complex public health data sets. To accomplish this objective, we investigated the factors associated with performance of TB screening of HIV-positive patients on basis of a large data set that was obtained at a Ghanaian HIV treatment facility. The next section deals with the theoretical mathematical framework of the LASSO and illustrates its practical implementation within a statistical framework using real data from the clinic in Kumasi, Ghana. The result section summarizes the cohort characteristics as well as the empirical findings. We conclude by discussing the study results and their implication as well as the methodological role and limitations of the LASSO procedure in public health data sets.

## Methods

### Data set

Between November 2011 and November 2012, adult HIV-positive patients consecutively presenting to the HIV outpatient department at the Komfo Anokye Teaching Hospital in Kumasi, Ghana, were enrolled into this study after written informed consent was obtained. The cohort comprised newly diagnosed, as well as HIV-positive patients already in care. The latter encompassed therapy-naïve individuals and patients on HAART. The present work was part of the HHECO study (NCT01897909) and approved by the appropriate ethics committees in Ghana and Germany. Demographical, socio-economical and clinical data were collected using a standardized questionnaire. Medical history and routine laboratory parameters were recorded from patient’s folders. Blood samples were obtained for analysis of CD4+/CD8+ T cell counts. As this study was conducted at a hospital within a so called “real life setting”, missing values in the data set occurred due to national stock outs of reagents needed for laboratory tests, patients forgetting to bring their test results, or other reasons. The cohort included a wide spectrum of HIV-infected patients regarding duration of HIV diagnosis and current status of antiretroviral therapy.

### Statistical method – the LASSO

The LASSO procedure is a shrinkage method within linear regression models that enables to shrink estimates of irrelevant variables towards zero and thus, allows for automatic variable selection. The LASSO combines the advantages of subset selection procedure (ease of interpretation) and ridge regression (robustness). In contrast to ordinary least squares (OLS), where estimates are obtained by minimizing the residual square error and therefore, tend to be overestimated although they are best linear unbiased estimators (1), penalized regression models minimize the regression coefficient by introduction of an auxiliary condition [14].

1 $$ {\widehat{\beta}}_{OLS}= \arg { \min}_{\beta}\left\{{\left|y-X\beta \right|}^2\right\} $$

In LASSO the auxiliary condition for β allows for penalizing the absolute size of the regression coefficient on basis of the tuning parameter value λ (2).

2 $$ {\widehat{\beta}}_{LASSO}= \arg { \min}_{\beta}\left\{{\left|y-X\beta \right|}^2+\lambda {\displaystyle \sum_{j=1}^P\left|{\beta}_j\right|}\right\}\kern1em  with\kern0.5em \lambda \ge 0 $$

The solution requires the LASSO estimates themselves shrinking towards zero. As a result, automatic variable selection is performed since only non-zero estimates have influence on the target variable. λ is a user-defined parameter that controls the amount of shrinkage, with increasing values of λ resulting in larger shrinkage. To choose the appropriate value of λ, cross-validation is the simplest and most widely used method [14–16].

Concerning the LASSO estimates, due to shrinkage the non-zero coefficients are generally biased towards zero. To reduce this bias, a common approach is to run the LASSO in a first step in order to identify the set of non-zero estimators and in a second step to fit an unrestricted model to the selected predictors. Alternatively, the LASSO can be applied in the second step again, but then using only the selected variables from the first step. Then, due to fewer variables and hence, less noise in the second run, λ is chosen with a smaller value. As a consequence, estimators will be shrunken less than in the first run of LASSO [15].

The LASSO is especially helpful in large data sets where efficient and fast algorithms are essential. Regarding the presence of multicollinearity among predictors, LASSO estimators are more stable and produce sparser solutions than those in OLS, where multicollinearity would lead to poor parameter estimates and hence to wrong inferences. However, since the LASSO penalty expects only a small set of coefficients to be non-zero or not close to zero, the method is not robust to high correlations among predictors as it will then quite arbitrarily choose one and drop the others. In this case, application of other variable selection procedures, such as elastic net, might be more suitable [17].

### Data analysis

Concerning the outcome variable, which states whether the TB screening has been done or not, a default in TB screening was defined if either a screening for TB has not been done or if an asserted screening was not documented in the patient’s folder. Among the outcome variable and predictors, high numbers of missing values occurred if some costly laboratory tests were not covered by the national AIDS programme and patients were not able to pay for these tests. Categorical variables with more than two categories were coded into dummy variables, whereby categories with the largest number of observations were defined as base category. For the missing values, the dummy coding method [18] was used for two reasons: First, deleting not fully observed cases or imputing missing values is impractical due to a high number of missing values. Second, many variables refer to laboratory test results and a missing value mostly indicates that the corresponding blood test has not been done for some reason. Therefore, missing values themselves carry important information as the outcome variable tells whether the TB screening has been done or not. The dummy coding method works as follows: For categorical variables, an extra category is built for missing values. For numeric variables, an extra dummy variable is created where 0 indicates an observed variable, whereas 1 indicates a missing observation. Subsequently, missing values in the numeric variable can be replaced by a constant value, e.g. 0. To address the high number of new HIV diagnoses coming to the clinic for the first time, we coded an extra dummy for this case.

**Fig. 1:**
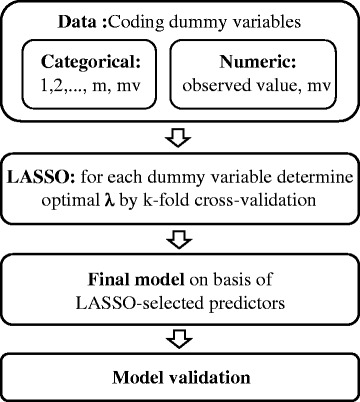
legend: m = number of variable categories; mv = missing values. Summary of the model construction steps

In order to identify relevant predictors, all explanatory variables available in the data set were entered into the LASSO procedure. The optimal penalty parameter λ was determined by 10-fold cross-validation. In a second step, shrinkage bias in selected non-zero coefficients was reduced by fitting logistic regression to the preselected predictors. The pseudo R-squared resulting from the logistic regression is not a reliable measure for the model validity because it ignores the variable selection step. In order to calculate a reliable measure for the model validity here, we used the conventional validation technique and therefore, randomly split the data into two data sets. The first data set contained 70 % of the data and was used as training data on which variable selection via the LASSO was done. The remaining 30 % were used as the test data on which the logistic regression and the corresponding pseudo R-squared were calculated. To reduce the random effect arising out of the random split of the data, this procedure was repeated 10 times and the average value of the obtained pseudo R-squareds was calculated. This procedure was only done for the calculation of the pseudo R-squared, all other results were obtained by applying LASSO and logistic regression on the whole data set. For summary of our model construction steps, see Fig. 1. Analyses were done by using Stata (version 10.1) and R software for statistical computing (version 3.1.2) using the glmnet package [19].

## Results

### Cohort characteristics

One Thousand Ninety Five HIV-positive patients were enrolled into this study. Three patients were excluded from the data analysis because information on the outcome variable was not available in the study data. At the time of recruitment, 690 participants (63.19 %) had performance of TB screening documented in their folder.

Participants without TB screening were diagnosed with HIV for a longer time period compared to those with documentation (mean 3.05 vs. 2.04 years, *p* < 0.001). They were more likely to be on antiretroviral therapy (*n* = 239 (59.45 %) vs. *n* = 291 (42.17 %), *p* < 0.001) and reported less often to use a mosquito bed net (*n* = 192 (47.88 %) vs. *n* = 421 (61.10 %), *p* < 0.001) compared to those for whom a TB screening was done and documented. The sub-groups did not differ in terms of age, gender, religion, education, occupation and socio-economic status (Table 1).

**Table 1:** Cohort characteristics

Variable	HIV-positive patients with TB screening	HIV-positive patients without TB screening	p-value
	*n* = 690 (63.19 %)	*n* = 402 (36.81 %)	
Gender, n (%)			0.070
Female	516 (74.78)	320 (79.60)	
Male	174 (25.22)	82 (20.40)	
Age in years, mean (± SD)	40.2 (9.71)	39.8 (9.09)	0.498
Receiving HAART, n (%)			<0.001
Yes	291 (42.17)	239 (59.45)	
No	399 (57.83)	163 (40.55)	
Years since diagnosis, mean (± SD)	2.04 (2.72)	3.05 (2.73)	<0.001
Religion, n (%)			0.751
Christian	590 (85.51)	345 (85.82)	
Moslem	87 (12.61)	50 (12.44)	
Traditional African religion	2 (0.18)	0 (0)	
Other	11 (1.59)	7 (1.74)	
Educational level, n (%)			0.056
No formal education	130 (18.84)	82 (20.4)	
Primary education	97 (14.06)	82 (20.4)	
Junior High School	321 (46.52)	168 (41.79)	
Senior High School	103 (14.93)	52 (12.94)	
Tertiary education	39 (5.65)	18 (4.48)	
Occupation, n (%)			0.468
Housewife	14 (2.03)	4 (1.0)	
Farmer	58 (8.41)	30 (7.46)	
Trader	347 (50.29)	225 (55.97)	
Salary worker	44 (6.38)	21 (5.22)	
Others	141 (20.43)	75 (18.66)	
Currently unemployed	86 (12.46)	47 (11.69)	
Access to tap water, n (%)			0.647
Yes	366 (53.04)	219 (54.48)	
No	324 (46.96)	183 (45.52)	
Electricity in the household, n (%)			0.676
Yes	639 (92.61)	375 (93.28)	
No	51 (7.39)	27 (6.27)	
Television in the household, n (%)			0.872
Yes	562 (81.45)	329 (81.84)	
No	128 (18.55)	73 (18.16)	
Owning a fridge, n (%)			0.484
Yes	491 (71.16)	278 (69.15)	
No	199 (28.84)	124 (30.85)	
Owning a car, n (%)			0.062
Yes	54 (7.83)	45 (11.19)	
No	636 (92.17)	357 (88.18)	
Using a mosquito bed net, n (%)			<0.001
Yes	421 (61.01)	192 (47.76)	
No	268 (38.84)	209 (51.99)	

*HAART* highly active antiretroviral therapy, *TB* Tuberculosis, *Without TB screening* Screening not done/not documented, p was determined by chi-square test or two-group mean-comparison *t*-test as appropriate

### Factors associated with performance of tuberculosis screening

As a result of coding dummies, the number of variables entering into the LASSO procedure increased from initially 101 to 156 variables. All parameters preselected via the LASSO, were included into the logistic multivariate regression model. Results show that unavailability of a test result for the HIV type (*n* = 305 (27.39 %), aOR 0.56, 95 % CI 0.41–0.77, *p* < 0.001), no information on the symptom of acute fever (*n* = 407 (37.27 %), aOR 0.511, 95 % CI 0.37–0.71, *p* <0.001), high haemoglobin (aOR 0.97, 95 % CI 0.95–1.0, *p* = 0.041) and a high CD4+ T cell count (aOR 0.92, 95 % CI 0.88–0.97, *p* < 0.001) are negatively associated with the performance of TB screening (Table 2).

**Table 2:** Factors associated with conduction of TB-screening – aOR from logistic regression

Dummy-variable chosen by LASSO	Number enrolledn [%]	Odds ratio	95 % CI	*P*-value
Time since HIV diagnosis (years)				0.003
> 0 (and not available)	703 [64.65] (32 [2.93])	1		
0	354 [32.42]	1.844	1.237–2.753	
HIV Type				<0.001
available	787 [72.07]	1		
not available	305 [27.93]	0.562	0.409–0.771	
CD4+ T cell count/(μl*100)	504 [46.15]	0.924	0.882–0.966	<0.001
Haemoglobin in g/dL	512 [46.89]	0.9734	0.949–0.999	0.041
Combination therapy with Lamivudine				0.858
yes	516 [47.25]	0.967	0.668–1.399	
no	576 [52.75]	1		
Information on acute fever				<0.001
available	685 [62.73]	1	0.369–0.707	
not available	407 [37.27]	0.511		
Chronic cough (past Six months)				0.013
yes	32 [2.93]	5.065	1.622–22.677	
no (and not available)	1,059 [96.98] (1 [0.09])	1		
Intake of regular medicine				0.661
yes	362 (33.15)	1.089	0.743–1.596	
no (and not available)	728 [66.67] (2 [0.18])	1		
Intake of co-trimoxazole				
yes	271 [24.82]	2.311	1.523–3.536	<0.001
no	821 [75.18]	1		
Supplementation with vitamins				<0.001
yes	220 [20.15]	2.641	1.722–4.101	
no	872 [79.85]	1		
Use of mosquito bed net				0.004
yes	613 [56.14]	1.535	1.149–2.0515	
no (and not available)	477 [43.68] (2 [0.18])	1		

Total number of observations = 1,092; *TB* Tuberculosis, Pseudo R-squared = 0.24

A positive association between predictors and the screening for TB could be observed for newly diagnosed HIV infections (*n* = 354 (32.42 %) aOR 1.84, 95 % CI 1.24–2.75, *p* = 0.003), chronic cough during the past six months (*n* = 32 (2.93 %), aOR 5.07, 95 % CI 1.62–22.68, *p* = 0.013), the intake of co-trimoxazole (*n* = 271 (24.82), aOR 2.31, 95 % CI 1.52–3.54, *p* < 0.001) or vitamin supplementation (*n* = 220 (20.15 %), aOR 2.64, 95 % CI 1.72–4.10, *p* < 0.001), as well as for the use of a mosquito bed net (*n* = 613 (56.14 %), aOR 1.53, 95 % CI 1.15–2.05, *p* = 0.004).

Although preselected by LASSO to be associated with conduction of TB screening, the parameters “Combination therapy with Lamivudine” and “intake of regular medicine” were not found to be significant predictors at the 5 % level in the final regression model. Finally, the measure of model validity pseudo R-squared was determined with a value of 0.24.

## Discussion

Screening HIV-positive patients for TB is essential for increasing the TB case detection rate and lowering the burden in PLHIV. Undetected cases of active TB are not only a common cause of death in HIV-positive individuals, but also a high risk for family members, hospital staff and fuel the expansion of multi resistant TB strains [20, 21]. To address shortages of staff and time, the WHO developed a TB screening form that simplifies integration of TB screening as a standard procedure in the clinical routine in resource-limited settings. With the help of the Global Fund to fight AIDS, Tuberculosis and Malaria, Ghana has put much effort into restructuring and integrating TB and HIV services within the public health system during the last years [22]. However, our data analysis indicates that there still is a high proportion of HIV-positive patients in Ghana, for whom TB screening has not been done or documented (36.81 %). These findings are in line with results from a survey conducted by the TB Control Programme in Ghana in 2013, in which the TB prevalence appears to be higher than the one estimated by the WHO [23]. This questions the estimated case detection rate of 88 %, that is likely to be overestimated [24]. Recent literature divides the underlying reasons for delay in TB diagnostics and treatment generally into health provider associated or patient associated factors [25]. The objective of this study was to identify factors related to performing of TB screening, which could serve as a contribution that enables public health decision makers to close these gaps and to increase the TB case detection rate among PLHIV.

According to our analysis, the odds of conduction and documentation of TB screening were much higher with newly diagnosed HIV infection (aOR 1.84) compared to those who were not newly diagnosed. This suggests that the WHO screening tool has been adopted as a standard procedure for new HIV cases like in many other sub-Saharan countries [26, 27]. But in order to reach fully successful implementation, screening of all cases has to be targeted [28].

Results also show that a high CD4+ T cell count and high haemoglobin, which indicate a good control of the HIV-infection, are negatively associated with TB screening, whereas chronic coughing during the past six months, which is a possible sign of active TB, is positively associated but does not trigger TB screening in every case. These findings demonstrate that TB screening is still related to current health status as it was practice before the WHO recommended TB screening as standard procedure regardless of the patient’s disease progress. A similar observation has been made in South Africa, where the burden of HIV/TB co-infection is high and the WHO stage was associated with delay in TB diagnosis and treatment [29]. Although it is advisable to screen patients with more advanced HIV stages more often, it should not replace general TB screening of every HIV case [30].

Evidence was found for a positive association between TB screening and usage of mosquito bed nets. This association did not appear for any other socio-economic parameter such as age, gender, religion, or educational level, so that usage of bed nets can rather be seen as a sign of patient’s health awareness. This finding is in accordance with another study conducted in Ghana in 2013 where Osei et al. found an independent inverse relationship between holding a health insurance and patient-associated delay in TB diagnostics, even though TB diagnostics and treatment are provided free of charge [31]. Also positively associated are preventive measures, such as vitamin supplementation and prescription of co-trimoxazole, as indicators for comprehensive medical care provided by clinical staff. However, missing information on current symptoms like acute fever or the unavailability of HIV type test results are negatively associated with screening for TB, which suggests that defaults in various preventive measures often go hand in hand. Several other studies have claimed that low quality or lack of records are not only harmful for individual patients because clinical or laboratory tests have to be repeated or are skipped. They state that missing data are likely to result in overestimation of case finding rates and therefore could lead to suboptimal decision-making in the public health sector [32–34].

## Conclusions

In order to address the LASSO’s limitation of selected estimates being shrunken and therefore biased we used the LASSO-method only for preselection of predictors. Thus, fitting an unrestricted linear model to LASSO-preselected predictors remains essential for further data analysis. Another limitation of this study is that data on patients’ and clinicians’ knowledge about TB screening was not collected. However, since this knowledge is likely to show a link to whether screening is conducted or not, further research on this topic should include additional behavioural factors.

Based on the results of this study the following conclusions can be offered:

- Patients should be counselled on individual and social benefits of preventive measures.
- Education and external monitoring of HIV clinic staff and the integration of TB screening into standard operation procedures is essential as screening activities are decisive for increased case finding.
- Sufficient financing of health systems has to be provided to avoid under-staffing in integrated services and stock outs of drugs and reagents as a potential reason for decreasing staff and patient motivation [35–37].
- Applying the LASSO method to our large public health data set was of crucial importance for identifying relevant predictor variables that were related to our outcome variable. Because the LASSO offers solutions to problems that are not addressed within conventional variable selection models, it proved as a valuable and efficient procedure for our analysis.

## Abbreviations

HAART, highly active antiretroviral therapy; HIV, human immunodeficiency virus; LASSO, Least Absolute Shrinkage and Selection Operators; OLS, ordinary least squares; PLHIV, people living with hiv; TB, tuberculosis; WHO, World Health Organization
